# Mitigating airborne pathogen risks in a full-scale meat processing facility

**DOI:** 10.1016/j.temicr.2025.100025

**Published:** 2025-07-16

**Authors:** Meiyi Zhang, Hyoungmook Pak, Stephen D. King, Alexander A. Zuniga, Yassin A. Hassan, Maria D. King

**Affiliations:** a Department of Biological and Agricultural Engineering, Texas A&M University, 333 Spence St, College Station, TX, 77843, USA; b Department of Mechanical Engineering, Texas A&M University, 202 Spence St, College Station, TX 77843, USA; c Department of Nuclear Engineering, Texas A&M University, 423 Spence St, College Station, TX, 77843, USA

**Keywords:** Bioaerosol, Meat processing facilities, Foodborne pathogens, qPCR, Metagenomics, Air curtains

## Abstract

Foodborne illnesses caused by Shiga toxin-producing *Escherichia coli* (STEC) and *Salmonella* represent a major public health concern, particularly in meat processing facilities where bioaerosols generated during processes like carcass spraying and dehiding can lead to contamination. In this study, we assessed airborne concentrations of STEC and *Salmonella* at multiple locations within a full-scale meat processing facility using quantitative polymerase chain reaction (qPCR) and Illumina MiSeq sequencing. Additionally, we utilized computational fluid dynamics (CFD) simulations to model airflow within the facility and evaluated the effectiveness of air curtains in mitigating the transfer of bioaerosols between high-risk (dehiding and tripe) and low-risk (chiller and fabrication) areas. qPCR results showed that pathogen concentrations in the dehiding rooms were 126 GCN/m^3^ for STEC and 105 GCN/m^3^ for *Salmonella* during spring, with levels rising significantly in summer (2198 GCN/m^3^ for STEC and 1799 GCN/m^3^ for *Salmonella*). Simulated airflow patterns revealed that entrained bioaerosols could be transported from unclean to clean areas, increasing the risk of cross-contamination. The use of air curtains effectively reduced this spread by creating barriers between high- and low-risk areas. Our findings suggest that bacterial survivability and aerosolization was enhanced in summer, highlighting the critical role of environmental factors and airflow management in controlling contamination risks. This study demonstrates the value of integrating experimental data with CFD simulations to assess pathogen spread and identify effective mitigation strategies in meat processing facilities.

## 1. Introduction

The presence of airborne pathogens in food processing environments, particularly in meat packing facilities, represents a significant public health risk to both workers and consumers. Beef production is one of the largest industries in the United States, generating about $100 billion in cash receipts in 2023 (USDA 2024). However, contamination events during meat processing can significantly contribute to the transmission of foodborne pathogens such as *Salmonella* spp. and Shiga toxin-producing *Escherichia coli* (STEC), which are frequently detected in meat products. The Centers for Disease Control and Prevention (CDC) estimated approximately 48 million cases of foodborne illness annually in the United States, with meat and poultry being the most common sources of fatal cases accounting for around 29 % of deaths (CDC 2018). From 2009 to 2015, out of the 2953 outbreaks that were caused by a single pathogen, 30 % were attributed to *Salmonella* and 6 % to STEC (Dewey-Mattia et al., 2018). Globally, *Salmonella* is responsible for an estimated 93.8 million illnesses and 155,000 deaths annually, while STEC causes around 2.8 million illnesses, highlighting the growing health concerns related to foodborne diseases in the meat processing industry (Heredia and García, 2018).

Pathogenic bacteria are introduced into meat products during the slaughter and processing of cattle in meatpacking facilities. A study conducted at a beef packing plant demonstrated that *E. coli* was detected on cutting tables, mesh gloves, and conveyor belts, with significantly higher contamination levels (*p* < 0.05) after work began compared to before (Yang et al., 2017a). During slaughter and skinning, cattle hides and feces are known to contaminate carcasses with pathogens such as *E. coli* and *Salmonella*, as well as fecal aerosols (Buncic et al., 2014). Mechanical actions during hide removal can generate bioaerosol droplets aerosolized droplets containing fecal matter, which may carry pathogenic bacteria through the air and deposit them onto carcasses (Schmidt et al., 2012). Bioaerosols, which are viable contaminants suspended on liquid droplets ranging from 0.1 μm to over 100 μm in diameter, can carry various microorganisms through the air, enabling them to travel significant distances and enhancing their survivability (Lutgring et al., 1997). Despite its significance for being a critical concern for food safety and hygiene, the spread of pathogenic bacteria via bioaerosols remains underexplored. However, this phenomenon has been observed in several meat processing plants, indicating a critical need for methods to track aerosolized pathogens at different locations within these facilities (Masotti et al., 2019). Several commercially available devices, such as impactors, impingers, and sedimentation sponges, are commonly employed to sample airborne particles (Haig et al., 2016). In this study, wetted wall cyclones (WWC) developed by McFarland et al. (McFarland et al., 2010) were utilized for bioaerosol collection, chosen for their capacity to preserve the viability of bacteria, large sampling volumes, and high collection efficiency (Zhang et al., 2025).

Understanding the factors that contribute to bioaerosol formation and spread is essential for mitigating pathogen transmission in meat processing plants. The concentration of bioaerosols in indoor environments varies based on environmental conditions, room function, and seasonal variations (Pak and King, 2022; Zhang and King, 2023; L. Zhang et al., 2022; Prussin et al., 2017; Ghosh et al., 2015). Several studies have demonstrated that temperature and relative humidity influence the concentration and stability of bioaerosols, thereby affecting their likelihood of resuspension in the air and subsequent deposition onto clean surfaces (Riddell et al., 2020; M. Zhang et al., 2022; Chai et al., 2023). Given the significant impact of environmental factors on bioaerosol behavior, it is crucial to explore effective methods for controlling and inactivating microorganisms to prevent their spread in meat processing environments.

Computational fluid dynamics (CFD), which employs the Navier-Stokes equations to model complex fluid flow phenomena, has been widely applied in critical indoor environment such as hospitals, classrooms, food processing facilities, and residential buildings to simulate airflow movement and predict pathogen transmission (Peng et al., 2020; Kumar and King, 2022; Kumar et al., 2023; Baig et al., 2022; Xia and Sun, 2002; Norton and Sun, 2006). Various methods exist for inactivating or removing microorganisms in bioaerosols, including dielectric barrier discharge plasma (Gallagher et al., 2007), carbon nanotube-based filters (Yang et al., 2017b), and HEPA filters (Brincat et al., 2016). The chosen solution for this study had to be cost-effective, easy to operate, and minimally invasive to the meat processing environment. An air curtain, typically installed at doorways, generates planes of air jets that create a barrier to block contaminants, heat, and smoke. This device effectively restricts air exchange between zones without interfering with the movement of personnel or meat carcasses. Experimental studies and numerical simulations have evaluated the performance of air curtains in various settings (Viegas et al., 2018; Frank and Linden, 2014; Foster et al., 2007). However, despite the extensive application of CFD in indoor environment modeling, there remains a significant gap in understanding how airflow interventions, such as air curtains, specifically influence bioaerosol dynamics in meat processing facilities.

The main objective of this study was to investigate the bioaerosol contamination at multiple locations in a meat processing facility across the spring and summer, focusing on total bacterial counts, *Salmonella*, and STEC in the air using quantitative polymerase chain reaction (qPCR). Additionally, a metagenomic analysis was performed to identify other relevant microbial taxa. CFD simulations was utilized to assess the effectiveness of air curtains as a mitigation strategy to limit bioaerosol spread across various locations within the facility. Few studies have investigated real-world airborne pathogens in full-scale, actively operating meat processing plants, limiting our understanding of bioaerosol dynamics in these complex, high-risk environments. This study aims to fill this research gap by integrating microbial detection and CFD simulations to provide insight into airborne pathogen dynamics and evaluate a practical mitigation strategy to reduce pathogen spread.

## 2. Materials and methods

### 2.1. Site description

**Fig. 1.**
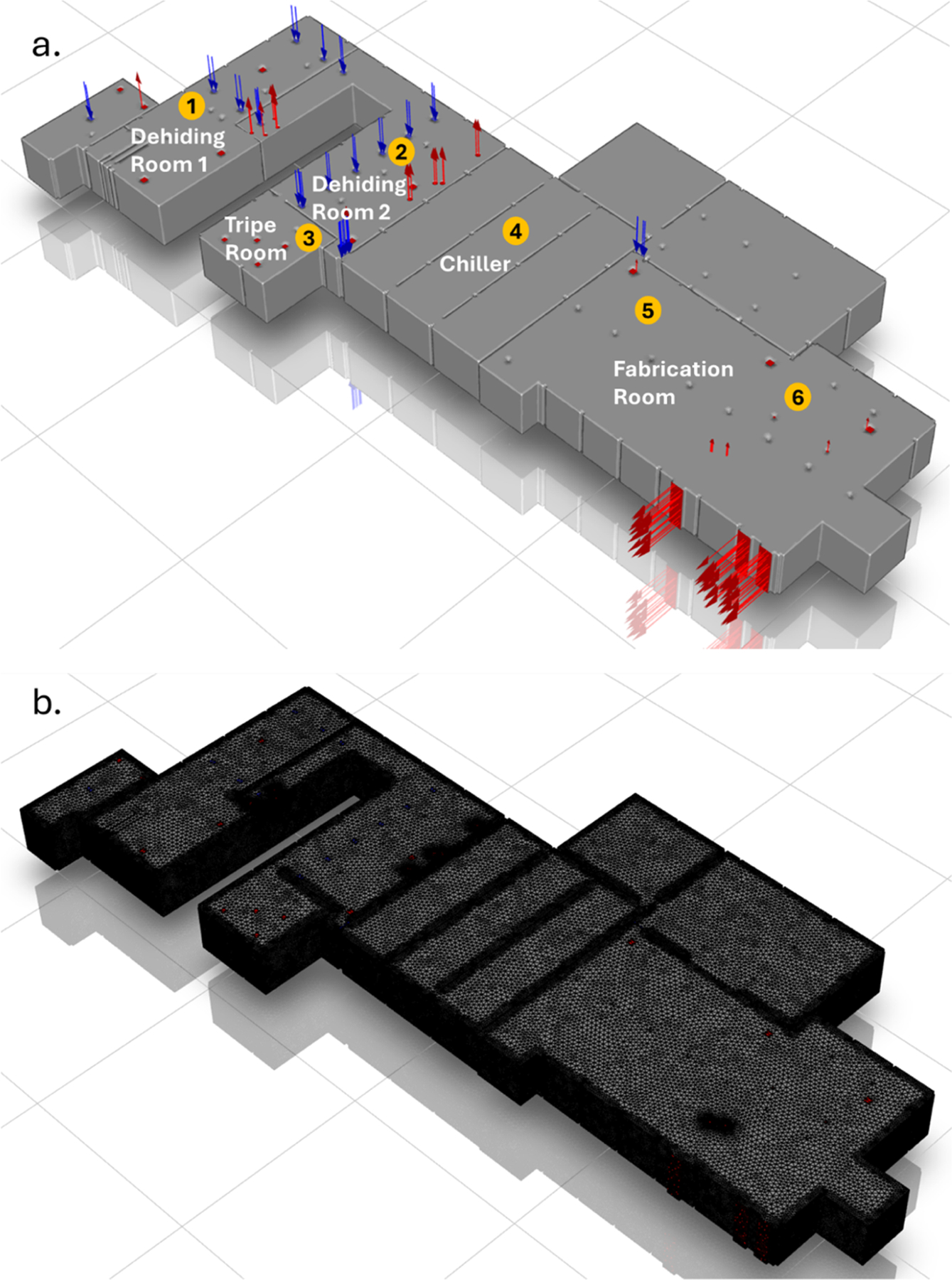
(a) Top view of Facility A. The numbers represent the locations where WWC units were set up to collect bioaerosol samples. (b) Surface mesh of the facility.

Two sampling campaigns were conducted to a full-scale meat processing facility, referred to as Facility A, during the spring and summer. The 12,542 m^2^ facility processes an average of 1800 cattle per day and performs key procedures, including skinning, evisceration, washing, chilling, and packaging. To examine the dynamics of aerosolized bacteria, WWC units were operated during working hours to collect air samples from various locations within the facility: dehiding rooms, tripe room, chiller, and fabrication room. The layout and the surface mesh of the rooms with the air inlet and exhaust locations and the bioaerosol collection sites in Facility A is shown in Fig. 1a and b. In the dehiding rooms, the WWC was initially set up at Site 1, but was later moved to Site 2 to capture a broader sample from the entire space. Similarly, in the fabrication room, the WWC was initially placed at Site 5 and then moved to Site 6 to cover a larger area. The dehiding rooms were slightly different in function: the first room is where cattle are knocked, exsanguinated, and skinned using automatic hide pullers, while the second room is dedicated to evisceration, splitting, and washing. The dehiding rooms and tripe room were categorized as the “unclean” area, while the chiller and fabrication rooms were considered the “clean” area. These locations were selected to assess how airflow influences bacterial spread throughout the facility.

### 2.2. Sample collection

Prior to each sampling trip, the WWC units were thoroughly cleaned with a 10 % bleach solution, followed by isopropanol, and then rinsed with sterile Milli-Q water. The system was flushed for 10 min to ensure any residue was removed. At the facility, air samples were collected at a rate of 100 L/min into 50 mL sterile Falcon tubes containing Milli-Q water, which were immediately placed on ice. Should the WWC aerosol testing include a culturability assessment, the collection tubes are spiked with 10 % phosphate buffered saline (PBS, pH 7.4) to provide osmotic support for the collected bacteria. Bioaerosol samples were collected in the morning and afternoon during each sampling campaign. However, since the tripe room only operated in the afternoon during the spring collection day, no samples were collected from the tripe room in the morning. Similarly, the tripe room operated in the morning in the summer sampling campaign, so no samples were collected from it in the afternoon. The WWCs operated during the facility’s four-hour work shifts, and the volume of air collected was recorded for each sample.

### 2.3. Temperature and relative humidity measurements

Temperature and relative humidity (RH) at sampling locations were continuously recorded every five seconds using HOBO data loggers (U12–013, Onset, Bourne, MA). These loggers were placed near the WWCs in the dehiding and fabrication rooms, where the highest levels of dynamic activity and environmental fluctuations occurred. The data loggers operated continuously from 8:30 AM to 6:30 PM during the sampling campaigns. The devices had an accuracy of ± 0.35 °C (± 0.63°F) for temperature and ± 2.5 % for relative humidity.

### 2.4. DNA extraction and sequencing

DNA extraction from the collected samples was performed using a previously described alkaline lysis method (Zhou et al., 1990). Bioaerosol samples were first centrifuged, and the resulting pellets were resuspended in 300 μL of TENS buffer. To the resuspended pellets, 150 μL of 3 N sodium acetate was added, followed by another round of centrifugation. The supernatants were carefully transferred to new Eppendorf tubes, and 10 μL of Poly Acryl Carrier (PAC, Molecular Research Center) was added to concentrate the nucleic acid. DNA was then precipitated by adding 1 mL of 100 % isopropanol to the samples. After centrifugation, the supernatants were discarded, and the pellets were washed with 1 mL of cold 100 % ethanol. Following an additional centrifugation step, the ethanol was removed, and the pellets were air-dried before being dissolved in 50 μL of sterile Milli-Q water. Extracted DNA was sent to the Texas A&M Institute for Genome Sciences and Society (TIGSS) for 16S rRNA sequencing, utilizing the Illumina MiSeq 2 × 250 v2 sequencing kit. The resulting sequences were analyzed using QIIME2 software and the SILVA database to identify the microbial taxa. Principal Coordinates Analysis (PCoA) was performed in R Studio to visualize differences in microbial composition in the samples, and Permutational Multivariate Analysis of Variance (PERMANOVA) was conducted to assess the statistical significance.

### 2.5. Real-time qPCR analysis

The sequences of the primers used to detect Total Bacterial Count (TBC), STEC and *Salmonella* are shown in Table 1. Virulence genes *stx* and *eae* were selected to identify the presence of STEC, and the *inv*A gene was targeted to detect *Salmonella* spp. in the bioaerosol samples (Singh et al., 2019; Bonetta et al., 2011). For gene amplification, 3 μL of extracted DNA was added to PCR tubes containing 1 μL of each forward and reverse primer, along with 5 μL of 2X SYBR Green PCR Master Mix (Applied Biosystems, Foster City, CA). Amplification was performed using the StepOne RT-PCR System (Applied Biosystems, Foster City, CA), with a melting curve analysis conducted within the temperature range of 60–90 °C. The thermocycling conditions included an initial denaturation step at 95 °C for 10 min, followed by 40 cycles of 95 °C for 15 s and 60 °C for 60 s. Threshold cycle (Ct) values obtained were used to calculate the concentration of aerosolized bacteria in terms of Gene Copy Number (GCN) per mL, based on the standard curves (King and McFarland, 2012). By incorporating both the sample volume and the volume of air collected, the GCN per cubic meter of air was then determined.

### 2.6. CFD simulation and air flow model

A solid model of Facility A was created using SolidWorks (SolidWorks 2019–20) and imported into ANSYS Fluent (ANSYS 2019 R2) for CFD analysis. The simulation aimed to assess the dispersion of pathogenic bacteria during meat processing. Certain elements, such as conveyor belts, were excluded to simplify the model and reduce computational complexity. The airflow data for Facility A’s HVAC system, provided by Environmental Technical Services, Inc., defined the inlet velocities in the CFD model. The dehiding rooms were equipped with 19 exhaust fans and 13 make-up air (MUA) units, while the fabrication room contained 7 exhaust fans. The MUA units were designated as velocity inlets, the exhaust fans were assigned as outlets, and the openings between rooms were modeled as pressure outlets.

Three different meshes were generated using tetrahedral cells: coarse (3,570,700 cells), medium (7,167,898 cells), and fine meshes (21,727,404 cells). The tetrahedral cells were then converted to polyhedral cells for the coarse (1,803,135 cells), medium (2,962,765 cells), and fine meshes (7,471,054 cells) to reduce the numerical diffusion and computing power requirement (Fig. 1b). Grid independence test was conducted to determine the optimal resolution where additional refinement did not significantly affect the simulation outputs. Among the three meshes, it was shown that the velocity profile stabilized between the medium and fine meshes, so the medium mesh with 2,962,765 cells was chosen for further simulation, which converged after 548 iterations.

Air flow simulations were performed under steady state condition using Reynolds Averaged Navier-Stokes (RANS) equations (ANSYS 2009). The realizable k-ε turbulence model was used to better account for turbulence and dispersion patterns and the standard wall function was used to account for turbulent flow prediction near the walls where 30 < *y*^+^ < 300. Prism layers were added to the walls, inlets, and outlets to satisfy the boundary layer requirements of the k-ε turbulence model. The SIMPLE algorithm and second-order upwind scheme were used for pressure, turbulent kinetic energy, turbulent kinetic dissipation, and energy. Residuals were set to 10^−6^ for energy and 10^−3^ for all others. Two air curtains were simulated in the CFD model: one between dehiding room 2 and the chiller, and the other between the chiller and fabrication room. Specifications of an Awoco 24″ Super Power 2 Speeds 800 CFM Commercial Indoor Air Curtain (Awoco, El Monte, CA) were used to simulate an average air velocity of 11.5 m/s. This simulation was conducted using the medium mesh under the same conditions and converged after 567 iterations. To validate the air curtain velocity profile, experimental measurements were taken at 30 locations in a test chamber (203 cm wide, 386 cm long, 206 cm high) using a VelociCalc hot-wire anemometer (TSI, Shoreview, Minnesota). These measurements were incorporated into the CFD model to accurately simulate airflow dynamics.

## 3. Results

### 3.1. Prevalence of total bacteria, STEC, and Salmonella in air using qPCR

**Fig. 2.**
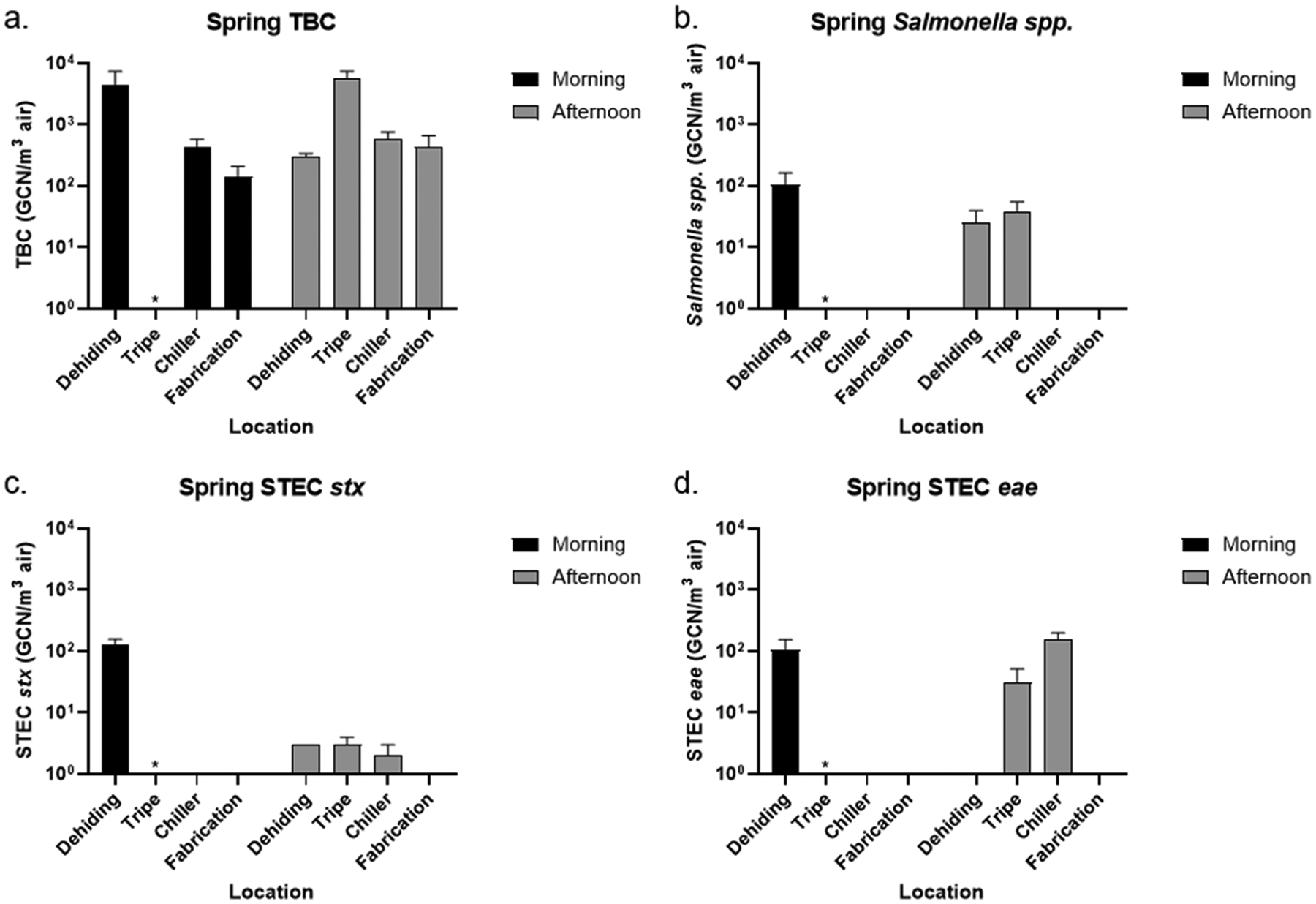
Concentration of (a) TBC, (b) *Salmonella*, and STEC carrying (c) *stx* and (d) *eae* in the air samples in the morning and afternoon at dehiding, tripe, chiller, and fabrication room during the spring collection day at Facility A. Detection of the bacterial species of interest was based on the presence of 16S, *inv*A, *stx*, and *eae* genes using qPCR. Sample was not collected from the tripe room in the morning (marked with asterisk) since the tripe room only operated in the afternoon on the collection day.

**Fig. 3.**
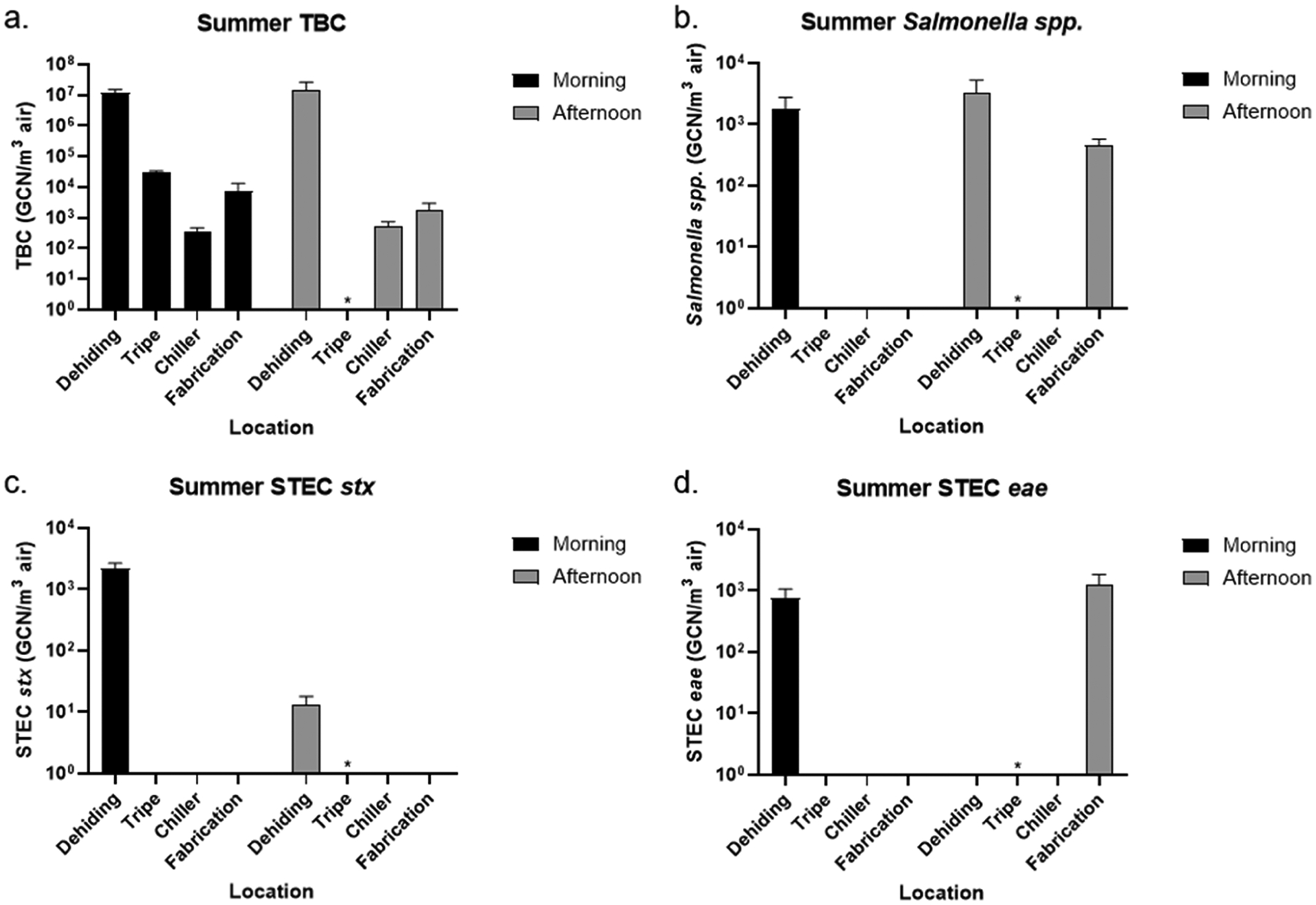
Concentration of (a) TBC, (b) *Salmonella*, and STEC carrying (c) *stx* and (d) *eae* in the air samples in the morning and afternoon at dehiding, tripe, chiller, and fabrication room during the summer collection day at Facility A. Detection of the bacterial species of interest was based on the presence of 16S, *inv*A, *stx*, and *eae* genes using qPCR. Sample was not collected from the tripe room in the afternoon (marked with asterisk) since the tripe room only operated in the morning on the collection day.

Air samples were collected from Facility A and analyzed with qPCR for detecting airborne bacteria of interest. Figs. 2 and 3 show the concentration of TBC, *Salmonella*, and STEC in the air in the morning and afternoon at four collection locations in the spring and summer based on the content of 16S, *inv*A, *stx*, and *eae* genes. The *inv*A gene encodes for a protein that is involved in the ability of *Salmonella* to invade the intestinal epithelial cells of the host, and is present in almost all *Salmonella* strains, making it a widely used and specific marker for identifying *Salmonella* (Galán et al., 1992). The *stx* gene encodes for Shiga toxin, a potent bacterial toxin that inhibits protein synthesis in host cells, leading to cell death (Melton-Celsa, 2014). The *eae* gene encodes for intimin, allowing adhesion to intestinal cells of the host (Zhang et al., 2021). As the *stx* gene confirms the Shiga toxin-producing potentials and the *eae* gene indicates the adhesion capabilities, both are used to identify the presence of pathogenic STEC strains. One important limitation of this study is that, because qPCR was used to detect the *stx* and *eae* genes, it cannot determine whether both genes originated from the same bacterial cell, even when both genes were present in a single sample. Therefore, while our results demonstrated the presence of these genes in the samples, we cannot confirm the presence of highly pathogenic STEC strains, which typically require both genes to be present in the same organism. Although previous studies have reported that both *eae*-positive/*stx*-negative and *stx*-positive/*eae*-negative *E. coli* strains can be associated with human illness, the majority of severe cases, particularly those leading to hemolytic uremic syndrome (HUS), are caused by strains that carry both genes (Beck et al., 2019; ANSYS 2019; Hua et al., 2020). As such, the public health risk inferred from our data must be interpreted cautiously within this methodological constraint. From our air collection in the spring, the highest total bacteria concentration was observed from the dehiding room in the morning at 4533 GCN/m^3^ air and from the tripe room in the afternoon at 5801 GCN/m^3^ air (Fig. 2a). The TBC in the chiller and fabrication rooms was about one order of magnitude lower in both the morning and afternoon compared to the highest total bacterial concentrations respectively. Our results suggest the hypothesis that cattle carcasses may be a source of bacterial contamination, as higher concentrations of aerosolized bacteria were observed in areas where carcass cutting and blood removal occurred, particularly in the connected dehiding and tripe rooms. Among the sampling locations, *Salmonella* spp. was identified at similar levels from the dehiding room throughout the day, and from the tripe room in the afternoon (Fig. 2b), which was directly connected to the dehiding room as shown in the facility layout in Fig. 1. As the tripe room only operated in the afternoon during the spring sampling day and was closed in the morning, the results indicate that the “unclean” area - dehiding and tripe rooms - were at higher risk of *Salmonella* spp. contamination compared to the “clean” area – chiller and fabrication room, although pathogen transmission from the unclean area seem to be less of a concern. Both the *stx* and *eae* genes were used in the analysis to identify STEC strains, as strains that contain both *eae* and *stx* genes possess the highest pathogenicity. While no viable STEC strains were isolated in this study, DNA from *eae*- and *stx*-positive bacteria was detected from the dehiding room in the morning and from the tripe and chiller rooms in the afternoon (Fig. 2c and d), indicating the presence of STEC without confirmation of live bacteria. Although STEC-associated genes were found at low levels in the afternoon, their presence in the chiller room may still pose a potential food safety risk, as cooler environments have been shown in previous studies to enhance bacterial persistence (Jones and Gibson, 2022; McEldowney and Fletcher, 1988). Across all locations and times, the dehiding room in the morning consistently showed higher concentrations of STEC. The lower airborne bacterial concentrations observed in the dehiding room in the afternoon can be attributed to the working schedule at Facility A, where the last animal is slaughtered by 3:00 PM, and meat cutting activities subsequently decrease in the dehiding room.

Similar qPCR analysis was conducted on the summer air samples. Fig. 3a shows that the highest total bacterial concentrations were recorded in the dehiding room, with 11,972,371 GCN/m^3^ air in the morning and 14,791,120 GCN/m^3^ air in the afternoon - approximately three orders of magnitude higher than those observed in the spring. Although TBC in the tripe, chiller, and fabrication rooms were significantly lower than in the dehiding rooms during the summer, the fabrication room had a TBC concentration about one order of magnitude higher in both the morning and afternoon compared to the spring (Fig. 2a). Regardless of the time of collection (morning or afternoon), the highest concentration of *Salmonella* spp. was detected in the dehiding room during the summer (Fig. 3b). Additionally, *Salmonella* spp. was present in the fabrication room in the afternoon at 460 GCN/m^3^ air, a location that tested negative in the spring. The airborne *Salmonella* levels in the summer (>460 GCN/m^3^ air) were significantly higher than those in the spring (<105 GCN/m^3^ air), indicating an overall increased risk of contamination. Although the *stx* gene was detected in the dehiding room in both the morning and afternoon, and the *eae* gene was detected in the dehiding room in the morning and fabrication room in the afternoon, only the morning dehiding room was both *eae*- and *stx*-positive (Fig. 3c and d). Similarly, STEC concentrations in the summer were much higher than in the spring overall.

### 3.2. Environmental conditions at facility A

**Fig. 4.**
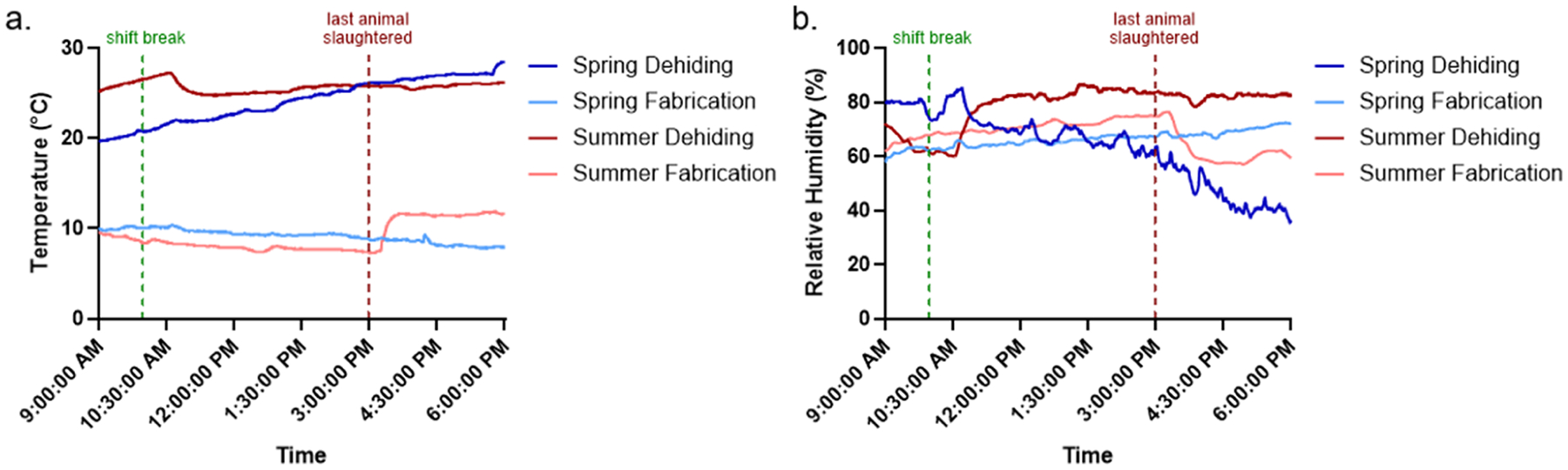
(a) Temperature and (b) RH measurements in the dehiding and fabrication rooms during the collection days in the spring and summer. Key facility events, including the morning shift break and the time of slaughter of the last animal, are indicated by vertical dotted lines in green and maroon, respectively.

Temperature and relative humidity (RH) were continuously monitored in the dehiding and fabrication rooms during the collection days in both the spring and summer seasons. Key facility events, including the morning shift break at 10:00 AM and the slaughter of the last animal at 3:00 PM, after which the HVAC system was turned off, were provided by facility management. On the spring collection day, the temperature in the dehiding room gradually increased from 20 °C at 9:00 AM to 28 °C at 6:00 PM, while the temperature in the fabrication room remained relatively stable, exhibiting a slight decrease from 10 °C to 8 °C (Fig. 4a). RH in the dehiding room showed the greatest fluctuation, decreasing from 80 % to 36 %, whereas RH in the fabrication room was more stable, increasing gradually from 60 % to 72 % (Fig. 4b). In contrast, during the summer collection day, the dehiding room temperature remained mostly stable with small fluctuations between 25 °C and 27 °C, while the fabrication room temperature gradually decreased from 10 °C to 7 °C before a sudden increase to 12 °C after the slaughter of the last animal (Fig. 4a). RH in the dehiding room showed a significant change, jumping from 60 % to 80 % shortly after the morning shift break, then stabilizing and fluctuating between 80 % and 85 % (Fig. 4b). In the fabrication room, RH gradually increased from 60 % to 76 % before suddenly dropping back to 60 % after the last animal was slaughtered. In the spring, temperature and RH showed an inverse correlation in both rooms, while in the summer, environmental conditions were more susceptible to facility events, leading to greater fluctuations. The increased instability and turbulence in the fabrication room during the summer likely contributed to the presence of airborne *Salmonella* and STEC, which were not detected in the spring (Figs. 2 and 3).

### 3.3. Microbial composition from 16S rRNA gene amplicon sequencing

**Fig. 5.**
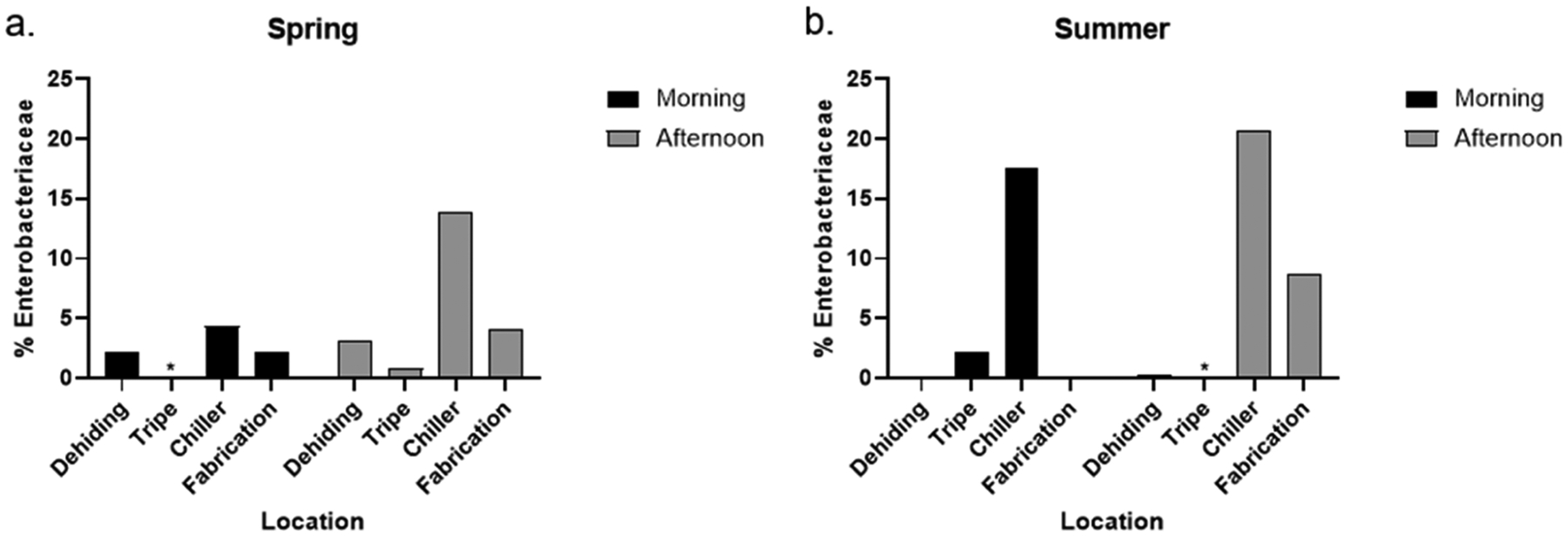
Percentage of sequences identified under the Enterobacteriaceae family level, out of the total sequences, at sampling locations in the morning and afternoon in (a) spring and (b) summer. STEC and *Salmonella* are both classified under the Enterobacteriaceae family level.

DNA extracted from air samples was analyzed using 16S rRNA Illumina sequencing, and microbial taxa were identified through the SILVA database. Negative controls including no-template PCR controls and DNA extraction blanks were included in the standard protocol to monitor for potential contamination. As 16S rRNA gene sequencing data are compositional, interpretations of microbial abundance are based on relative proportions and do not necessarily reflect absolute changes in microbial load. Fig. 5 shows the relative abundance of sequences classified under the Enterobacteriaceae family, where *Salmonella* and STEC are classified under. The Enterobacteriaceae family is a large group of bacteria commonly found in the intestines of humans and animals, including both harmless species and pathogenic species such as *Salmonella* and STEC (Octavia and Lan, 2014). The highest percentages of Enterobacteriaceae were observed in the chiller room, with values of 13.9 % in the spring and 20.7 % in the summer in the afternoon, followed by lower levels in the morning (4.4 % in the spring and 17.5 % in the summer). In the spring (Fig. 5a), the percentages of Enterobacteriaceae were similar in the dehiding room (2.1 % in the morning and 3.1 % in the afternoon) and fabrication room (2.2 % in the morning and 4.1 % in the afternoon). In contrast, during the summer, the fabrication room showed a notable increase in Enterobacteriaceae percentages, rising from 0.1 % in the morning to 8.7 % in the afternoon, likely due to processing activities (Fig. 5b). While the chiller room consistently exhibited high percentages of Enterobacteriaceae across both collection times and seasons, the total number of reads from sequencing was lower in this location, as shown in Table S1 in the Supplementary Materials. This suggests that the unique environmental conditions of the chiller room—such as lower temperatures, limited activity, and reduced air movement—may have led to a more stable microbial community compared to the more dynamic environments of the other rooms, which are subject to greater fluctuations in environmental conditions and processing activities.

**Fig. 6.**
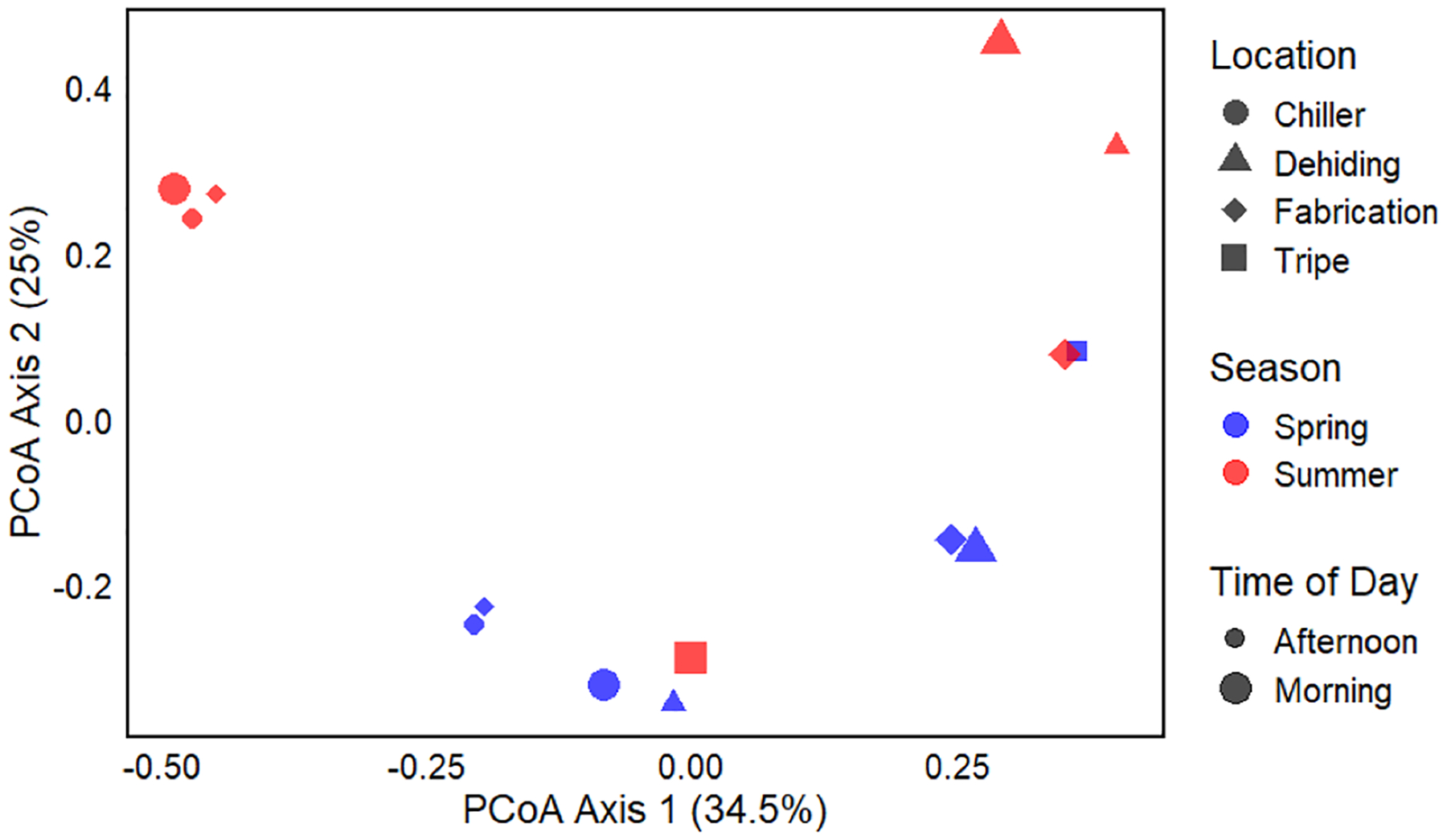
PCoA of microbial community composition of air samples based on the Bray-Curtis dissimilarity matrix, with points colored by season, shaped by location, and sized by time of Day.

To assess the overall differences in microbial community composition across sample groups, PCoA was performed using the Bray-Curtis dissimilarity matrix, based on the relative abundance of microbial taxa. Fig. 6 shows the PCoA plot with grouping variables such as season, location, and time of the day represented by different colors, shapes, and sizes. The PCoA revealed distinct clustering patterns in microbial composition based on location and time of day. In summer, the chiller and dehiding rooms formed tight clusters, both in the morning and afternoon, while the fabrication room in the morning of summer was positioned away from the other sample points. In spring, the dehiding and fabrication rooms in the morning overlapped, while all three locations – dehiding, chiller, and fabrication – in the afternoon, along with the chiller in the morning, clustered together. The similarity in microbiome compositions across all collection locations suggests that airborne bacteria may be able to travel between rooms in Facility A, potentially increasing the risk of cross-contamination. Notably, all spring samples were closely grouped, except for the tripe room, which was distantly located from other spring samples. In summer, the sample points were more dispersed, with the tripe room again located far from other summer samples. These findings indicate that the tripe room consistently exhibited a unique microbiome composition in both seasons compared to other locations. Additionally, microbiome compositions in the summer were more diverse compared to those in the spring. The chiller room microbiome composition was similar across both seasons, consistent with the results shown in Fig. 5.

To evaluate whether observed differences in microbiome composition were statistically significant, a PERMANOVA test was conducted. The results revealed that season (spring or summer) significantly influenced the microbiome composition (*p* = 0.021), explaining 16.2 % of the variation in microbial communities. While location (dehiding, tripe, chiller, or fabrication) showed a trend toward significance (*p* = 0.059), it accounted for 30.5 % of the variation. However, time (morning or afternoon) did not have a significant effect (*p* = 0.387), explaining only 5.9 % of the variation. These findings suggest that season and location are key determinants of microbial community structure, while time of the day appears to have a minimal impact.

### 3.4. Effectiveness of the air curtain control measure using CFD

**Fig. 7.**
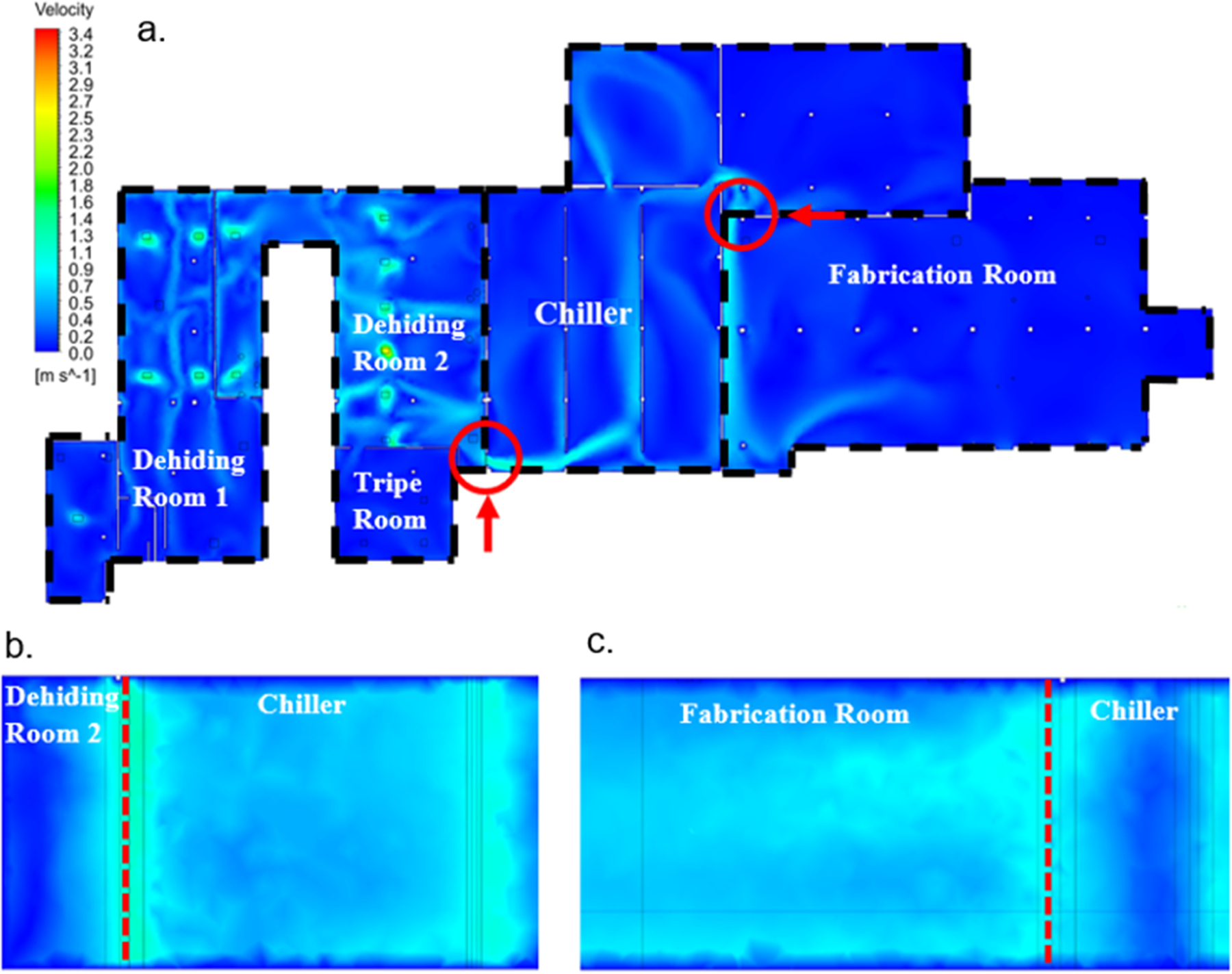
(a) Top view of the air velocity contour map across Facility A, with red circles indicating open passageways through which carcasses are conveyed. Arrows show the direction of the side views for each opening. (b) Side view of air velocities at the opening between dehiding room 2 and the chiller. (c) Side view of air velocities at the opening between the chiller and fabrication room. Dashed lines indicate the locations of the openings.

CFD simulations were conducted to evaluate the effectiveness of an air curtain as a control measure for reducing bacterial cross-contamination in Facility A. A contour map of air velocities (Fig. 7a) was used to visualize the airflow throughout Facility A. In the simulation, the MUA units located in the dehiding rooms were designated as air inlets, pushing air from these rooms toward the chiller and fabrication rooms. This airflow pattern reflects the movement of air through the facility, with the air being directed from the dehiding rooms, through the open passages, and into the adjacent areas, including the chiller and fabrication rooms. The facility can be divided into three main sections: the dehiding rooms 1 and 2 with the embedded tripe room, the chiller, and the fabrication room. Average air velocities were measured at 1.1 m/s between dehiding room 2 and the chiller, and 0.8 m/s between the chiller and fabrication room (Fig. 7b and c). These velocity profiles were nearly uniform from floor to ceiling. Without air barriers, Fig. 7a shows that air from the dehiding rooms, potentially containing entrained bacteria, dispersed into the chiller, contaminating surfaces where carcasses were stored, and further spread into the fabrication room. This finding aligns with qPCR analysis, which detected high concentrations of TBC in the dehiding and tripe rooms in spring, with smaller amounts found in the chiller and fabrication rooms (Fig. 2a), suggesting that airborne transmission occurred throughout the facility.

**Fig. 8.**
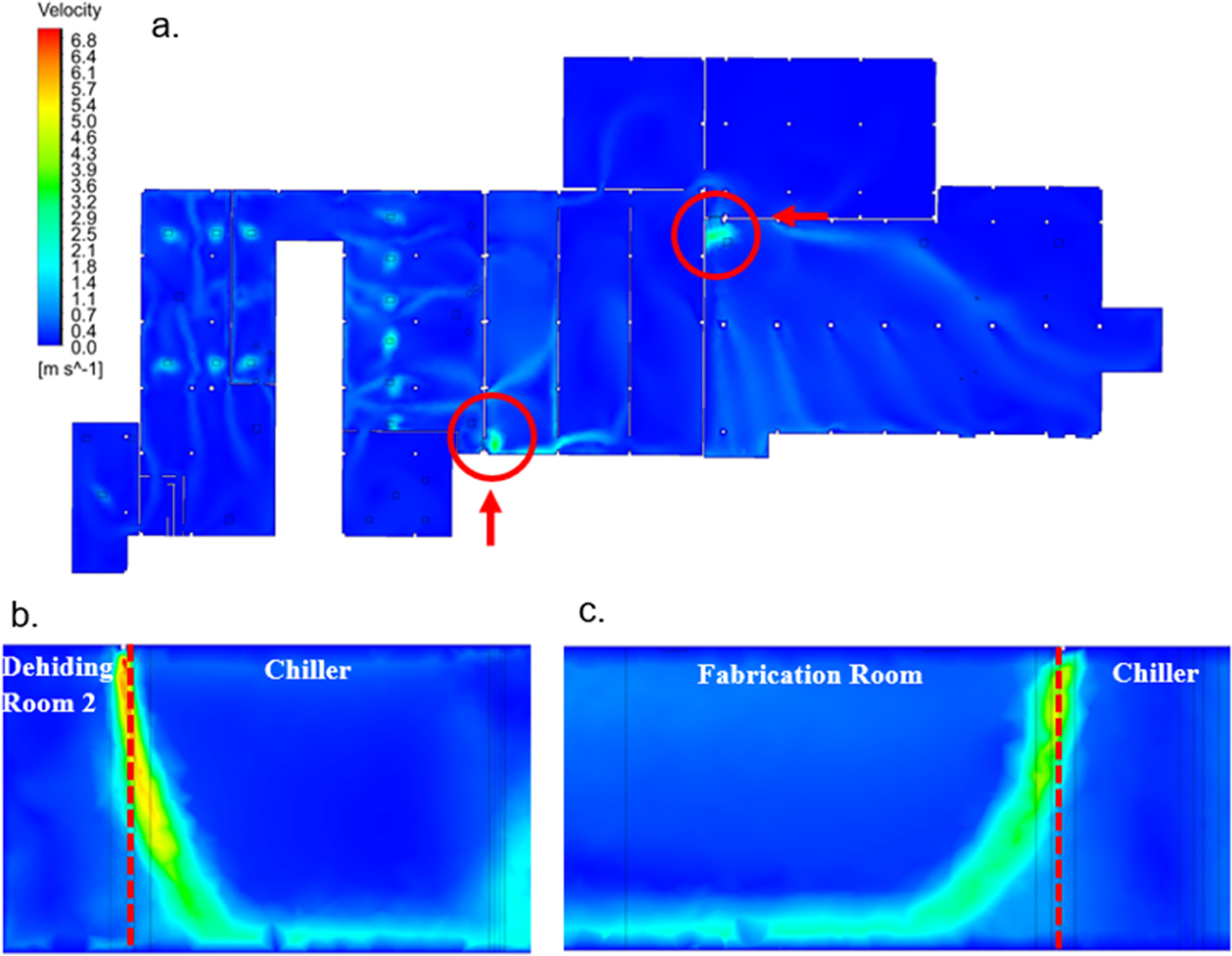
(a) Top view of the air velocity contour map across Facility A with the addition of air curtains at the openings, indicated by the circles. Arrows show the direction of side views for each opening. (b) Side view of air velocities at the opening between dehiding room 2 and the chiller, with air curtains installed. (c) Side view of air velocities at the opening between the chiller and fabrication room, with air curtains installed. Dashed lines indicate the locations of the openings.

To assess the impact of the air curtain, another CFD simulation was conducted with air curtains installed above the doorways. The air curtain inlets were set to an average velocity of 11.5 m/s. The contour map (Fig. 8a) shows areas of high air velocities where the air stream from the air curtain impacts the ground, creating a barrier that blocks airflow into the chiller and fabrication rooms (Fig. 8b and c). While air velocity within the facility nearly doubled compared to the baseline (without air curtains), the formation of these air barriers effectively prevented the spread of airborne contaminants into the clean areas.

To verify the CFD results, air velocity measurements were conducted in a model chamber with a commercial air curtain installed above the doorway. As shown in the Supplementary Materials, measurements were taken at 30 locations inside and outside the chamber (Fig. S1). The highest air velocities were recorded near the center of the air curtain inlet, with 3.34 m/s inside the chamber and 7.85 m/s outside (Fig. S2a and b). CFD results from the model chamber simulation (Fig. S2c and d) closely matched the experimental measurements, confirming that the airflow patterns were consistent. Additionally, a side view of the chamber (Fig. S3) confirmed the presence of a clear barrier formed by the air curtain, with recirculating eddies visible beneath the curtain, indicating effective containment of airborne contaminants.

These results demonstrate that air curtains can effectively modify airflow, creating barriers that mitigate the spread of airborne pathogens in meat processing facilities. The CFD simulations, together with experimental verification, suggest that air curtains are a promising control measure to reduce contamination risks in both the chiller and fabrication rooms of Facility A.

## 4. Discussion

Airborne bacterial concentrations were measured in the spring and summer at Facility A, focusing on *Salmonella* and STEC using qPCR. The dehiding room consistently emerged as the highest-risk area, showing the highest concentrations of TBC, *Salmonella*, and STEC in both seasons. This finding aligns with a previous study that identified meat cutting and blood removal as major sources of aerosolized bacterial contamination (Diyantoro and Wardhana, 2019). The high bacterial concentrations in the dehiding room can be attributed to the large amounts of bacteria released during these processes. This is in agreement with earlier studies that also highlighted the splitting area as a primary site of elevated airborne contamination in meat and poultry processing plants (Hall et al., 2013; Dobeic et al., 2011; Whyte et al., 2001; Rahkio and Korkeala, 1997). Environmental factors such as temperature and relative humidity have been shown to influence the deposition and transmission of bioaerosols (Chai et al., 2023; Dunklin and Puck, 1948; Huang et al., 2024; Wolkoff and Kjærgaard, 2007; Loveniers et al., 2024). In this study, bacterial concentrations in the dehiding room were significantly higher during the summer months compared to spring. This seasonal variation is likely due to the warmer temperatures in the summer, which promote increased bacterial aerosolization. Furthermore, the dehiding room, being less controlled than other areas in the facility, is more susceptible to external environmental factors (Shale and Lues, 2007). Consistently higher concentrations of TBC, *Salmonella*, and STEC in the summer across all locations further suggest that warmer conditions enhance bacterial survival and aerosolization, leading to an increased risk of contamination (Sajjad et al., 2024). These findings underscore the need for stricter control measures during the summer to mitigate bacterial spread within meat processing facilities.

In terms of microbial composition, the metagenomic analysis revealed that season significantly impacted the microbial composition in the air samples. The PCoA graph, along with the results of the PERMANOVA test, showed that season accounted for 16.2 % of the variation in microbial composition. This finding is consistent with previous studies, which have demonstrated that foodborne disease outbreaks exhibit a correlation with seasonal variations, typically reaching their peak during summer months (Naumova et al., 2007; Amin, 2002; Simpson et al., 2020). The clustering of data points from different rooms, particularly during the spring afternoon, indicated that microbial communities were very similar across locations at this time. This suggests that bioaerosols can easily spread between different areas within the ventilated facility, increasing the risk of cross-contamination. This is consistent with previous studies, which have shown that bioaerosols can propagate between rooms in indoor environments (Kumar et al., 2023; Sousan et al., 2022; Xu et al., 2024). Such findings underscore the need for enhanced control measures to prevent the movement of bioaerosols from high-risk areas, such as the dehiding room, into cleaner zones like the chiller and fabrication rooms. The risk of cross-contamination is particularly concerning, and it highlights the necessity of improved containment strategies, such as air curtains, to restrict the movement of airborne contaminants.

CFD simulations were used to evaluate the effectiveness of air curtains in controlling the spread of bioaerosols based on the airflow patterns. The simulations demonstrated that air curtains could significantly reduce the transmission of airborne bacteria between high-risk and cleaner areas by creating a barrier. This finding is supported by previous studies that demonstrated air curtains can effectively confine contaminants and reduce the spread of bioaerosols, particularly in controlled environments (Kumar and King, 2022; Baig et al., 2022; Wang et al., 2020). The airflow patterns generated by the air curtains were shown to effectively prevent the movement of bioaerosols into the chiller and fabrication rooms, offering a potential solution to minimize cross-contamination. These findings suggest that air curtains could be a viable intervention for reducing bacterial spread in areas where bioaerosols are a concern. However, further experimental validation in real-world settings is needed to assess the practical feasibility of implementing this control measure.

Despite the valuable insights provided by this study, some limitations must be considered. The relatively short sampling period may not fully capture the variability in environmental conditions that could influence bioaerosol concentrations. Long-term monitoring would provide a more comprehensive understanding of how seasonal changes impact bioaerosol dynamics. Additionally, this study focused primarily on *Salmonella* and STEC, but future research should explore a wider range of bacterial pathogens to better assess the overall microbial risk in such environments. Importantly, the use of qPCR-based detection and 16S rRNA gene amplicon sequencing has inherent limitations. These molecular methods do not distinguish between viable and non-viable bacteria, and are subject to amplification and sequencing biases that can affect the accuracy. Future studies incorporating culture-based methods would enhance understanding of the actual microbial risks associated with bioaerosols in such settings. A broader pathogen analysis, coupled with long-term surveillance, would contribute to refining bioaerosol control strategies and improving food safety outcomes.

## 5. Conclusions

The dehiding room consistently exhibited the highest bacterial concentrations, highlighting its high contamination risk. The seasonal variation in bacterial concentrations was evident, with higher levels observed during the summer months. The sequencing analysis revealed significant seasonal influence in microbial composition. The clustering of microbial communities in the spring afternoon samples indicated dispersal of bioaerosols across the facility, raising concerns about potential cross-contamination from high-risk areas such as the dehiding room. CFD simulations showed that air curtains can create an effective barrier to prevent airborne bacteria movement between high-risk and cleaner areas, offering a cost-effective control measure to reduce cross-contamination. Overall, this study highlights the influence of both season and location on airborne bacteria presence and provides support for implementing spatial and seasonal control measures to enhance food safety in meat processing environments.

## Supplementary Material

Zhang et al 2025 TEM Suppl Mat

Supplementary material associated with this article can be found, in the online version, at doi:10.1016/j.temicr.2025.100025.

## Figures and Tables

**Table 1:** Primer sequences for TBC, STEC, and *Salmonella* amplifications.

Primer Name	Sequence	Length (bp)	Reference
16S 1048	GTGSTGCAYGGYTGTCGTCA	146	(King and McFarland, 2012)
16S 1194	ACGTCRTCCMCACCTTCCTC		
*stx* F	TTTGTYACTGTSACAGCWGAAGCYTTACG	131	(Perelle et al., 2004)
*stx* R	CCCCAGTTCARWGTRAGRTCMACDTC		
*eae* F	CATTGATCAGGATTTTTCTGGTGATA	102	(Nielsen and Andersen, 2003)
*eae* R	CTCATGCGGAAATAGCCGTTM		
*invA* F	GTGAAATTATCGCCACGTTCGGGCAA	284	(Rahn et al., 1992)
*invA* R	TCATCGCACCGTCAAAGGAACC		

## Data Availability

Data will be made available on request.
