# Supplementary material for: Mitigating airborne pathogen risks in a full-scale meat processing facility
Source: Total Environ Microbiol. Author manuscript; Available in PMC 2026 Mar 24. (PMC13008310; doi:10.1016/j.temicr.2025.100025)
Supplement: Zhang et al 2025 TEM Suppl Mat [file NIHMS2155589-supplement-Zhang_et_al_2025_TEM_Suppl_Mat.docx]

**Supplementary Materials**

**Table S1.** Percentages of Enterobacteriaceae sequences out of the total sequences obtained via Illumina sequencing of bioaerosol samples in Spring and Summer.

|  |  | Spring | | |  | Summer | | |
| --- | --- | --- | --- | --- | --- | --- | --- | --- |
| Time | Location | Enterobacteriaceae reads | Total  reads | Percentage |  | Enterobacteriaceae reads | Total  reads | Percentage |
| Morning | Dehiding | 1276 | 59485 | 2.1 |  | 33 | 398247 | 0.01 |
|  | Tripe | N/A | | |  | 339 | 15824 | 2.1 |
|  | Chiller | 652 | 14900 | 4.4 |  | 294 | 1680 | 17.5 |
|  | Fabrication | 928 | 42102 | 2.2 |  | 45 | 54322 | 0.1 |
| Afternoon | Dehiding | 533 | 17289 | 3.1 |  | 213 | 93089 | 0.2 |
|  | Tripe | 664 | 78770 | 0.8 |  | N/A | | |
|  | Chiller | 1348 | 9711 | 13.9 |  | 467 | 2261 | 20.7 |
|  | Fabrication | 447 | 10984 | 4.1 |  | 196 | 2261 | 8.7 |


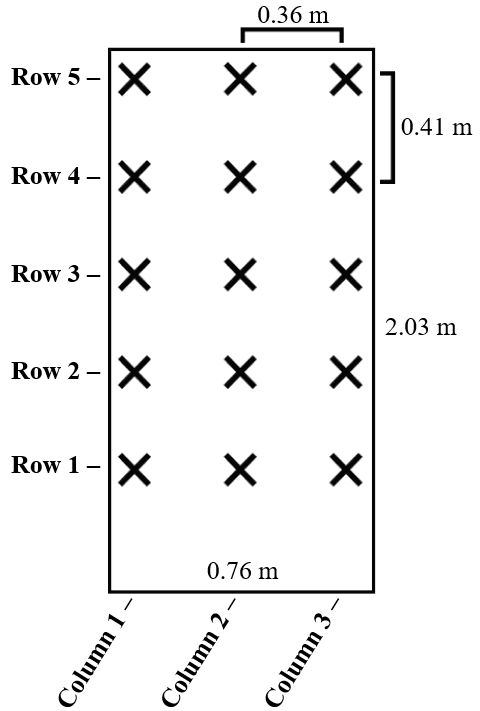


**Figure S1.** Locations of air velocity measurements taken to verify CFD results in a model chamber with a commercial air curtain. Fifteen measurements were recorded 0.127 m inside the chamber and fifteen measurements 0.127 m outside, relative to the air curtain inlet. The figure is not to scale.


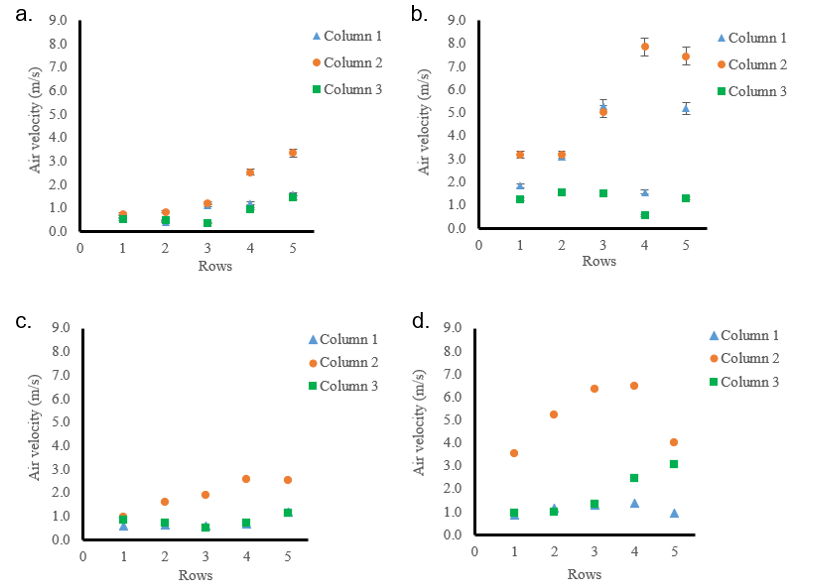


**Figure S2.** Air velocity measurements taken in the model chamber with a commercial air curtain. (a) Experimental measurements inside the chamber door, (b) experimental measurements outside the chamber door. CFD simulation results for air velocities (c) inside the chamber and (d) outside the chamber, corresponding to the experimental setup.


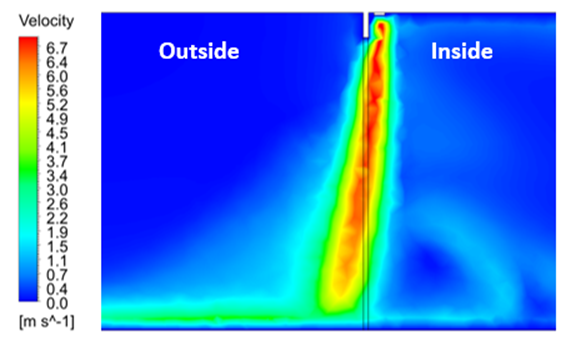


**Figure S3.** Side view contour map of air velocities at the model chamber door with the commercial air curtain installed, showing the formation of an air barrier and recirculating eddies beneath the curtain.
